# Eco‐Friendly Innovation: Biodegradable and Oil‐Resistant Bags From *Lotus halophilus* Extract, Polyvinyl Alcohol, and Guar Gum for Sustainable Food Packaging

**DOI:** 10.1002/fsn3.71017

**Published:** 2025-09-26

**Authors:** Osama Magouz, Eman Fayed, Khadija S. Radhi, Fayez Althobaiti, Ahmed Aboueloyoun Taha, Reham M. Kamel, Lamiaa I. El‐Nawasany, Mohamed Abdin, Diaeldin Omer Abdelkarim, Yahya S. Hamed, Mahmoud Younis

**Affiliations:** ^1^ Department of Food Science The Pennsylvania State University University Park Pennsylvania USA; ^2^ Department of Biotechnology, College of Sciences Taif University Taif Saudi Arabia; ^3^ Department of Science and Nutrition, College of Science Taif University Taif Saudi Arabia; ^4^ Soils, Water and Environment Research Institute, Agriculture Research Center Giza Egypt; ^5^ Agricultural Engineering Research Institute, Agricultural Research Center Giza Egypt; ^6^ Dairy Chemistry Department Animal Production Research Institute (APRI), Agricultural Research Center Giza Egypt; ^7^ Agricultural Research Center Food Technology Research Institute Giza Egypt; ^8^ Department of Agricultural Engineering, Faculty of Engineering University of Khartoum Khartoum Sudan; ^9^ Food Technology Department, Faculty of Agriculture Suez Canal University Ismailia Egypt; ^10^ Chair of Dates Industry and Technology King Saud University Riyadh Saudi Arabia

**Keywords:** biodegradable films, food packaging, *Lotus halophilus* extracts, PVA‐guar gum

## Abstract

Clear or improperly designed packaging can allow nonconvenient conditions to degrade the oil, leading to rancidity and loss of flavor. Biodegradable and effective packaging solutions are essential to maintaining the quality of olive oil and extending its shelf life. This study aimed to examine the production of biodegradable small sachets utilizing a blend of polyvinyl alcohol (PVA), Guar Gum (GG), and pure extract of *Lotus halophiles* (LHE). The study assessed these films' color, mechanical, thermal, and crystallinity characteristics, and their physical and surface shape. According to the findings, adding LHE up to a 1% concentration produced an opaque hue and raised the opacity to 4.132. Furthermore, the films exhibited reduced moisture content (9.87%), solubility (18.71%), water contact angle (WCA) (84.1%), and water vapor permeability (*WVP*) (2.121 × 10^−10^ g.m^−1^ s^−1^ pa^−1^). Incorporating 1% LHE led to smoother film surfaces, while higher concentrations (1.5% of LHE) resulted in rougher surfaces. Fourier transform infrared (FT‐IR) analyses verified that the PG matrix and the LHE extract physically interacted. The films also demonstrated excellent thermal stability; adding LHE enhanced their storage capacity to maintain olive oil quality.

## Graphical abstract

Biodegradable PVA–guar gum films with *Lotus halophilus* extract show enhanced antioxidant, antibacterial, and barrier properties for food packaging.

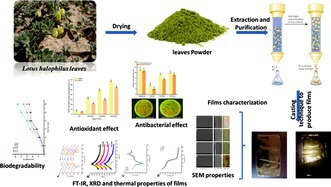

## 1 Introduction

The packaging industry, particularly in the food sector, often relies on petroleum‐based polymers due to their affordability, lightweight nature, long‐lasting durability, flexibility, and excellent mechanical, thermal, and barrier qualities (Sun et al. 2017). However, the global production of plastic surpasses 380 million tons, significantly damaging marine ecosystems and wildlife. The packaging industry contributes to about half of the globe's plastic pollution, primarily due to the extensive usage of virgin plastics with a limited lifespan (Vidal et al. 2022).

Converting petroleum‐based products to biodegradable alternatives is currently in its confirmation stage. Extensive research has been performed to find cost‐effective substitutes for petro‐based plastics with biodegradable properties. One approach is the development of biodegradable films using natural sources like starch‐derived polysaccharides, gums, natural fibers, chitosan, and cellulose extracted from animal sources. These approaches aim to reduce costs while promoting sustainable alternatives to traditional plastics (Priyadarsini et al. 2022). Among natural and biodegradable materials, polyvinyl alcohol (PVA) is a synthetic polymer with hydrophilic qualities, making it an excellent choice for packaging. PVA is a great material for packaging since it is colorless, nontoxic, and has excellent film‐forming qualities. It is commonly used as a bioplastic and exhibits impressive durability when subjected to different organic solvents. Furthermore, PVA is highly suitable for applications such as paper adhesives and packaging materials (Gómez‐Aldapa et al. 2020; Haghighi et al. 2020).

Guar gum (GG) is a refined nonionic polymer derived from the endosperm of the annual legume plant known as Indian cluster beans ( *Cyamopsis tetragonoloba* ). This polysaccharide, known as galactomannan, has a mannose‐to‐galactose ratio of 1:6. It consists of branch points of (1–6)α‐D‐galactopyranose and a (1–4) β‐D‐mannopyranose backbone, which are connected to galactose side groups (Nandi and Guha 2018). GG is an edible, abundant, and economically viable biocompatible, hydrophilic polysaccharide carbohydrate. It possesses excellent viscosifying power, approximately 5–8 times more effective at thickening than starch. These properties make it highly well‐suited for packaging uses, as it is nontoxic and biodegradable (Valencia et al. 2019). Extensive research has focused on developing composite films by blending synthetic and biopolymers to address the limitations of biopolymer‐based films, particularly their low mechanical strength and high susceptibility to environmental conditions (Saraiva et al. 2023). GG, as a reducing agent, and PVA, as a capping as well as stabilizing agent, were utilized via an in situ approach to form based nanocomposite films with a different weight ratio of silver nanoparticles as promising food packaging materials (Gasti et al. 2021).

Polymer blending is widely acknowledged as a highly efficient method for producing novel materials with customized features. When polymers are mixed to produce films, the outcome of the process often displays distinct mechanical and physical characteristics that differ from films created from individual polymers. Combining natural and synthetic polymers makes improving the overall worth of films feasible due to the easy accessibility and comparatively economical nature of synthetic polymers (Xu et al. 2023). Researchers are currently studying incorporating active agents to improve packaged meals' quality, safety, and efficiency. Plant extracts are widely favored as active ingredients in films because of their abundant phenolic components, which possess potent antioxidant capabilities.

For instance, a one‐pot green fabrication method for chitosan (CS)/PVA/Ag nanocomposite (CPAg) films, using *Spondias pinnata* fruit pulp extract (SPFPE) as a silver ion reducing agent, with the CS/PVA matrix, was used as both a capping and stabilizing agent for meat preservation (Gasti, Dixit, Shastri, et al. 2025). Additionally, PVA‐based halochromic films integrated with Na‐montmorillonite clay (MMT) and anthocyanin from *Ipomoea cairica* (IC) (Morning glory) flowers were prepared as a biosensor to monitor the freshness of lamb meat (Gasti, Dixit, Chougale, and Masti 2025). With more than 18,860 species and 630 genera, the Fabaceae family is regarded as the third‐largest family of varied species. It is the most common family in dry woods in America, Africa, and tropical rainforests, especially prevalent in medicinal plants (Xu et al. 2017).

*Lotus halophilus* , commonly called Sand Restharrow, is a yearly herb belonging to the Fabaceae family. It is indigenous to the Mediterranean region, Iran, and the Arabian Peninsula. This plant possesses compound, broad leaves and has a self‐supporting growth habit. While typically reaching a height of 6–12 cm, *Lotus halophilus* is considered a herbaceous perennial. It can be found in a variety of habitats, including tropical rainforests and arid woodlands in both America and Africa. Notably, this plant is renowned for its medicinal properties and is particularly abundant in phenolic compounds known for their powerful antioxidant qualities. The growing demand for high‐quality olive oils poses a challenge in developing packaging materials that can preserve the properties of Extra Virgin Olive Oil (EVOO) for a prolonged period. The packaging is essential for protecting the oil from the harmful effects of oxygen and light, which can directly affect its quality (Xu et al. 2017). The novelty lies in using *Lotus halophilus* extracts as functional additives in forming active packaging materials. Specifically, integrating these extracts into PVA and GG‐based films represents a pioneering approach to enhancing the preservation of olive oil quality during storage. Furthermore, *Lotus halophilus* , a plant known for its bioactive compounds, provides a fresh and sustainable source of antioxidants and functional properties that can effectively inhibit oxidative degradation in olive oil. Additionally, combining PVA and GG with plant extracts creates biodegradable packaging that serves as a physical barrier and actively contributes to preserving the oil's quality.

## 2 Materials and Methods

### 2.1 Materials

Fresh olive oil was procured from a local market. Fresh aerial parts of *Lotus halophilus* were collected in May 2022 (spring) from Wadi Habis, Matrouh Governorate, Egypt. Botanical identity was authenticated by the Botany Department, Faculty of Science, Cairo University, using the Flora of Egypt and comparison with Desert Research Center (DRC) herbarium specimens. To conduct the investigation, PVA (CAS No: 9002‐89‐5), having an average molecular weight of 30,000–70,000 (87%–90% hydrolyzed), along with guar gum (GG) with a viscosity of 30–150 cP, a 1% aqueous mixture (84%–95% hydrolyzed) was obtained from Gamma Scientific Company, Egypt. The DPPH, ABTS, peptone, and agar nutrients were procured from Sigma Aldrich Co. Ltd. in St. Louis, MO, USA.

### 2.2 Methods

#### 2.2.1 Production of Purified *Lotus halophilus* Leaves Extract (LHE)

The leaves were dehydrated by natural evaporation in a shaded area, subsequently dried in a heated oven at 40°C until they reached a stable mass. Ultimately, they were reduced to a tiny powder. The procedure described in reference Abdin et al. (2019) was used to obtain purified LHE. In summary, the process involved immersing 30 g of fine powder in 1 L of 70% ethanol and subjecting it to ultrasonic waves generated by a device operating at 1100 W and 220 V for 1 h. The end product was filtered under vacuum and then centrifuged at 1000 *g* for 10 min. The extract obtained after centrifugation was freeze‐dried at −50°C, resulting in a lyophilized powder. After lyophilization, the powder was diluted in purified water and applied onto a 5 × 30 cm D101 resin column. Following the rinsing of the solution with three bed volumes of filtered water, it was subsequently eluted using two bed volumes of 80% ethanol. Following purification, the extract was subjected to lyophilization at a temperature of −50°C and collected in a conical flask.

#### 2.2.2 LC‐ESI‐QTOF‐MS Analysis of Purified LHE

The extracted constituents of the LHE were identified using the LC‐ESI‐QTOF‐MS technique on a G2‐XS QTOF Waters instrument from Manchester, UK. To reach an amount of 2 mg/mL, the freeze‐dried extract was mixed in 70% ethanol and centrifuged at 2000 rpm for 20 min at 5°C. A 2 mL portion of the resulting supernatant was collected and put into HPLC vials for LC–MS analysis. Subsequently, a 2 μL volume of 0.1% formic acid dissolved in water (buffer A) and 0.1% formic acid in acetonitrile (buffer B) was employed as the mobile phases in the UPLC column. The software was designed to progressively decrease the concentration of buffer A from 80% to 5% over 40 min. This was achieved by reducing the flow rate of buffer A by 0.8 mL/min every 4 min. A vessel power of 3 kV was utilized, along with a sample cone voltage of 30 V. The temperature was sustained at 130°C, while the inert gas temperature was established at 700°C.

#### 2.2.3 Preparation of PG/LHE Sachets

First, 3 g of PVA were solubilized in 100 mL of distilled water at 95°C for 30 min while constantly mixing at 700 rpm. After that, 1 g of GG was combined with 150 mL of purified water in a different 500 mL beaker and heated to 50°C for an hour while agitated at 500 rpm. The PG mixture was formed by transferring the mixed PVA solution from the first beaker to the second beaker and stirring it for 1 h at a temperature of 60°C. During the ongoing stirring process, 1.8 g of glycerol was introduced, and various levels of LHE (0.5%, 1%, and 1.5% of the total mixture volume) were included to produce PG/LHE1, PG/LHE2, and PG/LHE3, respectively. Subsequently, an exact amount of 80 mL from the mixture was carefully transferred into petri plates with a 15 cm diameter. The petri plates were carefully positioned on an even surface in an air oven dryer, which was adjusted to 60°C and left for 7 h. After completely drying the films, they were meticulously extracted from the petri plates and positioned in a desiccator for more testing.

#### 2.2.4 Characterization of Surface and Cross‐Section

The microstructure of the films was assessed using scanning electron microscopy (SEM). The procedure below was used to prepare PG/LHE samples for SEM examination. The films were first dried after being cut into tiny square pieces. These square sections were subsequently adhered to metal grids with double‐sided adhesive tape for stability. Afterward, the samples' surface and cross‐section were coated with gold particles using a spray technique. Lastly, SEM, Philips‐FEI Co., AMS, Eindhoven, Netherlands, was used to take pictures of the produced samples. The SEM was configured for 500 μm power magnification and ran at a 5‐kV voltage.

#### 2.2.5 Fourier Transform Infrared Spectroscopy (FT‐IR)

The FT‐IR properties of films were characterized by utilizing the Nicolet 6700 FT‐IR spectrometer (Thermo Fisher Scientific Co. Ltd., Waltham, MA, USA). The samples were chopped into pieces of an appropriate size so that they would line up with the films' detector fields. After that, these samples were analyzed with a spectrometer that had an 8 cm^−1^ resolution and a cross‐spectral spectrum ranging from 500 cm^−1^ to 4000 cm^−1^.

#### 2.2.6 XRD Analysis of PG/LHE Films

Utilizing a Bruker D8 Advance X‐ray diffractometer (USA), XRD (X‐ray diffraction) patterns were tested to provide insights into the crystallization behavior of PG/LHE films. The scattered radiation was quantified within a predetermined range of 2*θ* = 5°–80° with the instrument running at 50 mA and 50 kV.

#### 2.2.7 DSC and TGA Characterization of PG/LHE Films

Differential scanning calorimetry (DSC) profiles for the films were generated following the established approach, utilizing a SHIMADZU DSC‐60 plus machine. A precise weight of 3 mg of the film sample was then moved into an aluminum pan, which was subsequently completely sealed using a piston. The sealed pans and empty reference pans were cautiously positioned within the machine. The temperature was then programmed to increase steadily from 0°C to 400°C at 10°C per unit time. This process allowed for the creation of DSC curves to analyze the thermal characteristics of the films.

The TGA analysis was conducted using the TGA‐50 thermogravimetric analyzer from Shimadzu, Japan. A weight of 10 mg of the sample was carefully transferred to a steel pan made specially to withstand high pressure. An empty pan was used as a point of reference to make a comparison. Subsequently, the machine was configured to incrementally increase to 600°C by employing a controlled temperature ramp of 10°C/min.

#### 2.2.8 Mechanical Characterizations of PG/LHE

The films were initially trimmed to 2 × 10 cm^2^ dimensions and securely positioned within the grips of Instron 5567 Co., USA. To ensure stability during the testing process, the device was set up with an initial distance between the grips of 50 mm and a cross‐head rate of 130 mm/min. The objective of this procedure was to calculate the films' TS (tensile strength) and EB (elongation at break) numbers, which are significant for assessing their durability and capacity to endure stretching without cracking. The pertinent Equations (1) and (2) were immediately employed to calculate the TS and EB values as described previously (Abdin et al. 2024)

(1) $$\mathrm{TS}\;\left(\mathrm{MPa}\right)=\frac{\mathrm{Fm}}{\mathrm{Th}\times \mathrm{Wi}}$$

(2) $$\mathrm{EB}\;\left(\%\right)=\left(\frac{L-L0}{L0}\right)\times 100$$

The term Fm refers to the highest‐level tension (N) that arises during stretching, where the terms stand for film thickness, wi for width, IL for film elasticity (mm), and L0 for starting grip film length (mm).

#### 2.2.9 Determination of Water Contact Angle (WCA) for PG/LHE Films

Using the drop methodology, a HARKE‐SPCAX1 optical contact angle (HARKE, China) measurement instrument was utilized to determine the WCA of PG/LHE films (Kraisit et al. 2015). Static water contact angle (WCA) was measured by the sessile‐drop method. Film specimens were secured flat on glass microscope slides with adhesive tape. A small droplet of distilled water was gently placed on the film surface, and its profile recorded. Four independent droplets were analyzed per sample at nonoverlapping locations to ensure reproducibility; mean ± SD was reported. Lower WCA denotes higher surface wettability of the film layer.

#### 2.2.10 Determination of Biodegradability of PG/LHE Films

The potential biodegradation of the PG/LHE films was assessed using the accepted approach outlined in Abdin et al. (2023). The films were placed 5 cm deep in a flower pot and continuously misted with daily sprinkler irrigation to maintain consistent soil moisture. After 30 days, the soil moisture content was measured at 37.5%. The films were removed and analyzed every 10 days to assess film degradation and weight loss. This measurement technique was conducted three times for accuracy, and the average was computed to ensure reliable findings.

#### 2.2.11 Antioxidant Activity of PG/LHE Films

The film samples weighing 0.025 g each were submerged in 3 mL of purified water to remove the components for further examination. The dissolution of the extract was facilitated through this immersion process. In order to assess the constituents for subsequent examination, the film samples, each weighing 25 mg, were dissolved in 3 mL of purified water. The breakdown of the mixture was expedited using this immersion method. To evaluate the scavenging activity of DPPH free radicals, we utilized an adapted iteration of a previously published method (Zhang et al. 2016). To describe the percentage of discoloration, a mixture was created by mixing 3.0 mL of the extracted solution containing 1.0 mL of a methanol mixture with 0.1 mM DPPH. The resultant combination was kept in a lightless setting for 30 min. After the incubation phase, the absorbance of the combination was quantified at 517 nm. The proportion of discoloration was evaluated utilizing subsequent Equation (3):

(3) $$\text{DPPH radical scavenging activity}\;\left(\%\right)=\left(\frac{\mathrm{Abs}\;\text{control}-\mathrm{Abs}\;\text{films}}{\mathrm{Abs}\;\text{control}}\right)\times 100$$

As mentioned, the procedure for determining the ABTS radical scavenging by PG/LHE films was conducted (Kim et al. 2018).

The ABTS radical cation solution was generated for the scavenging activity experiment by incubating a mixture of 145 mM potassium persulfate and 7 mM ABTS in darkness for 12 h. Following incubation, the solution's absorbance was adjusted to less than 0.8 using 0.2 M pH 7.4 phosphate‐buffered saline (PBS) to optimize assay sensitivity. To evaluate the free radical scavenging capacity of the film extract, 20 μL was combined with 1980 μL of the produced ABTS mixture. The resulting solution was then subjected to absorbance at a wavelength of 734 nm. The values obtained from this measurement were calculated using the following Equation (4):

(4) $$\text{ABTS radical scavenging activity}\;\left(\%\right)=\left(1-\frac{\mathrm{Abs}\;1-\mathrm{Abs}\;2}{\mathrm{Abs}\;0}\right)$$

Abs 1; the absorbance of ABTS samples is represented by Abs 0, and A2 represents the absorbance of the PBS sample.

#### 2.2.12 Antibacterial Efficacy of PG/LHE Films

The disc diffusion test assessed the samples' antibacterial capacity (Kaczmarek‐Szczepańska et al. 2021). The experiment employed two bacterial strains, *Salmonella typhimurium ATCC 14028* and *Clostridium botulinum sporogenes NCA 3679*. A 20 mL volume of nutritional agar broth with a pH range of 6.8–7.2, peptone (4.5 g/L), containing peptone (4.5 g/L) and yeast concentrate (2.5 g/L) was introduced into a conical flask for bacterial preparation. The flask was then treated for 24 h at 37°C with the bacterial strains. Following the incubation time, the bacterial cell suspension was spun up at 6000 × *g* for 2 min, and the supernatant was disposed of. After being cleaned twice with sterile NaCl solution (0.85%), the optical density of the remaining bacterial cells was set to 0.5 on the McFarland scale. Subsequently, sanitized circular film segments of 10 × 10 mm were positioned on petri plates filled with a solid substrate. Following the even spreading of 0.1 mL of bacterial culture over the medium, the dishes were set up for 24 h at 37°C. A sliding caliper was used to assess the inhibition zone that resulted during incubation.

#### 2.2.13 Assessment of Film Thickness, Moisture Content, Solubility, and Swelling Degree

The thickness of the PG/LHE films was measured using a micrometer. Before measurement, the films were cut into smaller sections and placed vertically between the anvil and spindle of the micrometer. Measurements were taken at six different positions across the film surface to ensure accuracy, with the micrometer held firmly in contact with the material. The average thickness was then calculated from the collected values.

The water solubility, moisture content, and swelling degree of the PG/LHE films were evaluated following the methodology outlined by Eltabakh et al. (2021). To determine the initial wet weight (M1), five film samples measuring 2 × 2 cm^2^ were weighed. These samples were then placed in a convection oven at 105°C until a constant weight was achieved. After drying, the final weight (M2) of the samples was recorded to assess the moisture‐related properties. The drying period was maintained at 37°C until weight stabilization occurred.

The moisture content was determined using Equation (5).

(5) $$\text{Moisture content}\;\left(\%\right)=\left(\frac{M1-M2}{M1}\right)\times 100$$

After dehydration, the samples were immersed in 30 mL of filtered water for a full day. The insoluble components of the films were kept by putting the liquid onto pre‐weighed filter paper. After that, the filter sheets were split into two categories: a consistent weight was attained by repeatedly drying a few filter papers at 105°C.

The final mass (M3) was determined by subtracting the initial weight from the final weight. The remaining filter papers were air‐dried at ambient temperature, precisely at 25°C, and the paper weights were documented (M4). The following formulas (6, 7) were used for the mentioned measurements;

(6) $$\text{Solubility}\;\left(\%\right)=\left(\frac{M2-M3}{M2}\right)\times 100$$

(7) $$\text{Swelling degree}\;\left(\%\right)=\left(\frac{M4-M3}{M3}\right)\times 100$$

#### 2.2.14 Characterization of Water Vapor Permeability (*WVP*)

The *WVP* of the PG/LHE films was assessed following the procedure described by Du et al. (2021). Initially, 15 mL of distilled water was added to a conical flask. A piece of PG/LHE film was tightly affixed over the mouth of the flask using a string to prevent vapor loss. The prepared flasks were then placed inside a desiccator maintained at a temperature of 25°C and a relative humidity of 50%.

The mass of the flasks was measured every 2 h for a total of 12 h, and the *WVP* was measured using a specific Equation (8).

(8) $$\mathrm{WVP}=\frac{\Delta w.f}{A.t.\Delta P}$$

The formula incorporates several variables to calculate the *WVP*. These variables include Δ*w*, indicating variations in the flask weight over duration; *f*, denoting film thickness; A, representing the exposed area of the PG/LHE film; *t*, signifying the time interval of approximately 2 h; and Δ*P*, indicating differences in water pressure.

#### 2.2.15 Characterization of Color Properties

The color and opacity properties were determined, as previously indicated (Abdin et al. 2024), with appropriate changes. The samples' L*, a*, and b* characteristics were evaluated using the Konica Minolta Cr color reader. Increases in a* and b* show that the samples are getting more red and yellow, respectively, while increases in L* show that the samples are getting more transparent. Before readings from four distinct sample sections were taken to determine the mean, the samples were put on the whiteboard of the machine and allowed to stabilize. Next, we used a particular equation to calculate the color difference, Δ*E* (Equation 9).

(9) $$\Delta E=\sqrt{{\left(\Delta L*\right)}^{2}+{\left(\Delta a*\right)}^{2}+{\left(\Delta b*\right)}^{2}}$$

The opacity of the films was assessed using a Genesys 10S UV–Vis spectrophotometer (Thermo Fisher Scientific, USA). Before analysis, the thickness of each film sample was measured and incorporated into the calculation of opacity at 600 nm, according to Equation (10) below.

(10) $$\text{Opacity}=\frac{\mathrm{Ab}600}{\text{Thickness}\;\left(\mathrm{mm}\right)}$$

#### 2.2.16 Small Sachets Applied as Active Packaging for Olive Oil

Olive oil was packaged in small sachets to be conveniently consumed in tiny portions. The PG/LHE2 films were selected because they had the best mechanical, surface structure, and *WVP* characteristics, and satisfactory antioxidant and antibacterial properties. Films were sliced and heat‐sealed on three ends to make 6 cm × 4 cm small sachets. After eliminating any air bubbles and adding 20 mL of olive oil, the sachets were heat‐sealed on top to create four‐sided sealed sheets. In contrast, the olive oil was also packaged in pouches composed of polypropylene film (PP film), readily available in the market. During preservation, the samples were exposed to particular acceleration conditions. This involved using a Luxometer V&A Instrument (model MS6610, China) to reveal the samples to fluorescent illumination with an intensity ranging from 900 to 1000 lx while keeping the temperature at 40°C ± 2°C.

#### 2.2.17 Characterization of Peroxide Value, K_232_ , and Acidity

The subsequent methodology was employed to ascertain the peroxide value (PV). Initially, 2 g of butter was combined with a solution containing acetic acid and chloroform at 12:18. Subsequently, 1 mL of a potassium iodide mixture at its maximum solubility was introduced into the combination. After 1 min of agitation, the resulting mixture was placed in a lightless environment for 5 min. The solution underwent titration with 0.01 N sodium thiosulfate after adding 30 mL of filtered water and 1 mL of starch solution. The PV measurement was performed using the provided formula (Equation 11).

(11) $$\mathrm{PV}=\frac{\left(V1-V2\right)*N*1000}{W}$$

The value of PV can be calculated with the formula as follows: V1 denotes the volume of sodium thiosulfate, V2 signifies the volume of sodium thiosulfate, *N* indicates the standard deviation or amount of the sodium thiosulfate solution, and *W* indicates the mass of the sample.

The K_232_ extinction coefficient was determined by measuring the absorption of a solution containing 1% alcohol in cyclohexane at 232 nm utilizing a UV spectrophotometer (UV‐1800, Shimadzu, Japan) with a path length of 1 cm. This measurement was conducted following the previous method (Cecchi et al. 2010). The acidity was characterized using the previous procedure (Marand et al. 2021).

A combination of 50:50 ethanol/chloroform was made, and titration was used to neutralize it. To do this, 3–4 drops of a 1 g/100 mL ethanol phenolphthalein solution were added until the liquid turned pink. Then, 5 g of the butter sample was put in an Erlenmeyer flask, and 25 mL of the produced chloroform/ethanol combination was poured in. Three to four drops of phenolphthalein reagent were introduced to the combination, and then a 0.01 N potassium hydroxide (KOH) solution was used for titration. The acidity was then determined using the following Equation (12):

(12) $$\text{Acidity}=\frac{\mathrm{KOH}\;\text{volume}*\mathrm{Mw}\;\text{of}\;\mathrm{KOH}*\text{normality of}\;\mathrm{KOH}}{\text{Weight of sample}}$$

#### 2.2.18 Determination of the Release of Film Components to Olive Oil Sachets

The release of polyphenolic compounds from the biodegradable films was indirectly determined by measuring the residual total phenolic content (TPC) within the film matrix over the 8‐day storage period. Film sachets containing a known volume of olive oil were prepared and stored in the dark at 37°C. At predetermined time intervals (Days 0, 2, 4, 6, 8), triplicate sachets were sacrificed. The olive oil was carefully decanted, and the film specimens were thoroughly rinsed with a hexane solution to remove any residual surface oil, followed by air‐drying to remove the solvent. The dried film samples were then cut into small pieces, and the polyphenols were extracted from the film matrix using 70% ethanol 70% for 6 h. The TPC of the resultant extract was quantified using the Folin–Ciocalteu method. The amount of polyphenols released was calculated by subtracting the residual TPC in the film at each time point from the initial TPC measured in the film before oil contact (Day 0), with results expressed as micrograms of gallic acid equivalents (GAE) per gram of film. This data was then used to model the release profile and rate of polyphenol migration.

#### 2.2.19 Statistical Analysis

Statistical analysis was conducted with SPSS 20.0 in order to identify variation. Tukey's HSD analysis was employed to detect statistically significant disparities among means. A *p* value below 0.05 was deemed statistically noteworthy.

## 3 Results and Discussion

### 3.1 Analysis of Purified LHE

Table 1 displays the outcomes of the chromatographic characteristics and mass spectrometry data examination of the nine substances shown. Among the chemicals provisionally identified, the predominant ones include ferulic acid, genistein, gallic acid, and astragalin, which were detected at concentrations of 71.42, 61.71, 43.36, and 42.16 μg/g of dry sample, respectively. The presence of genistein was proved in *Fabaceae* family plants (Das et al. 2020). Additionally, ferulic acid and astragalin, kaempferol, and gallic acid were proven in plants from the *Fabaceae* family (Obistioiu et al. 2021). Ferulic acid from plant resources is widely recognized for its potent antioxidant activity and its various benefits, including antimicrobial, anti‐inflammatory, and neuroprotective effects (Antonopoulou et al. 2022).

**TABLE 1:** The composition of bioactive compounds in the analyzed LHE by LC‐ESI‐QTOF‐MS.

Identified compounds	Retention time	MW (Da)	Calibration formula	Coefficient of determination *R* ^2^	μg/g of dry sample
Ferulic acid	16.86	194.25	γ = 5342.73*x* + 1381.6	0.9568	71.42
Vanillic acid	9.84	168.15	γ = 6534.60*x* + 5310.8	0.9986	22.09
Tamarixetin	11.24	316.99	γ = 6833.11*x* + 5313.7	0.9981	39.15
Astragalin	8.25	448.99	γ = 8543.17*x* + 4351.5	0.9792	42.16
Kaempferol	10.13	286.23	γ = 8432.62*x* + 5670.2	0.9760	11.85
Penocembrin	22.17	256.25	γ = 8252.83*x* + 5130.2	0.99860	39.17
Genistein	24.16	270.24	γ = 7342.25*x* + 4532.2	0.99786	61.71
Quercetin	14.25	302.96	γ = 6282.73*x* + 4252.3	0.9687	33.57
Gallic acid	6.83	170.12	γ = 7532.29*x* + 3652.4	0.9414	43.36

### 3.2 Film's Surface Structure by SEM Analysis

**FIGURE 1:**
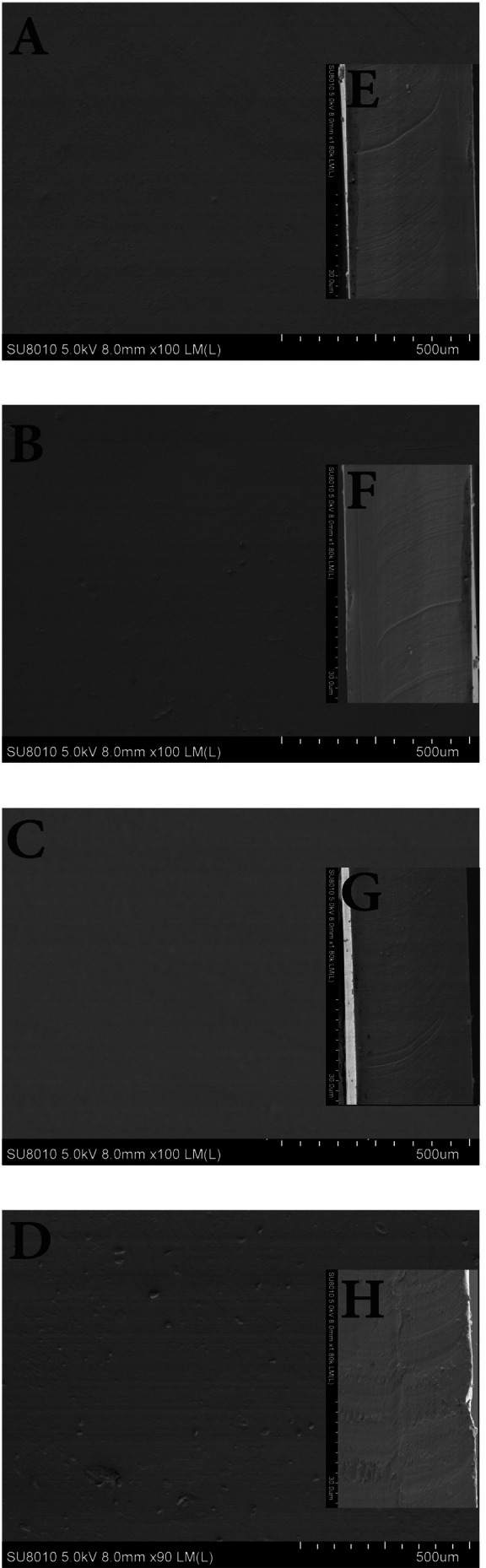
SEM micrographs of PG (A), PG/LHE1 (B), PG/LHE2 (C), PG/LHE3 (D), cross‐sectional images of PG (E), 308 PG/LHE1 (F), PG/LHE2 (G), PG/LHE3 (H).

SEM was used to examine the surface (Figure 1A–D) and cross‐sectional (Figure 1E–H) morphologies of the PG and PG/LHE and control films. The absence of apparent fissures on the smooth surface (Figure 1A) of the control films (PG) indicates that the polymers utilized were uniformly distributed and demonstrated molecular compatibility. There were no discernible changes at the lowest concentration of LHE when the surface and cross‐sectional morphology of PG films containing varying amounts of pure LHE were examined (Figure 1A–C). This implies that LHE phenolic compounds at lower concentrations promoted a positive interaction with PG films. In contrast, introducing larger concentrations (2.1%) of LHE to the PG films caused the films' cross‐sectional shape to exhibit obvious fissures, an uneven surface, and curls (Figure 1D). One reason for the inconsistency seen following integration with high levels of LHE could be the emergence of localized microphase separation within the PG matrix. The same behaviors were noted earlier (Abdin et al. 2023).

### 3.3 Films FT‐IR Analysis

**FIGURE 2:**
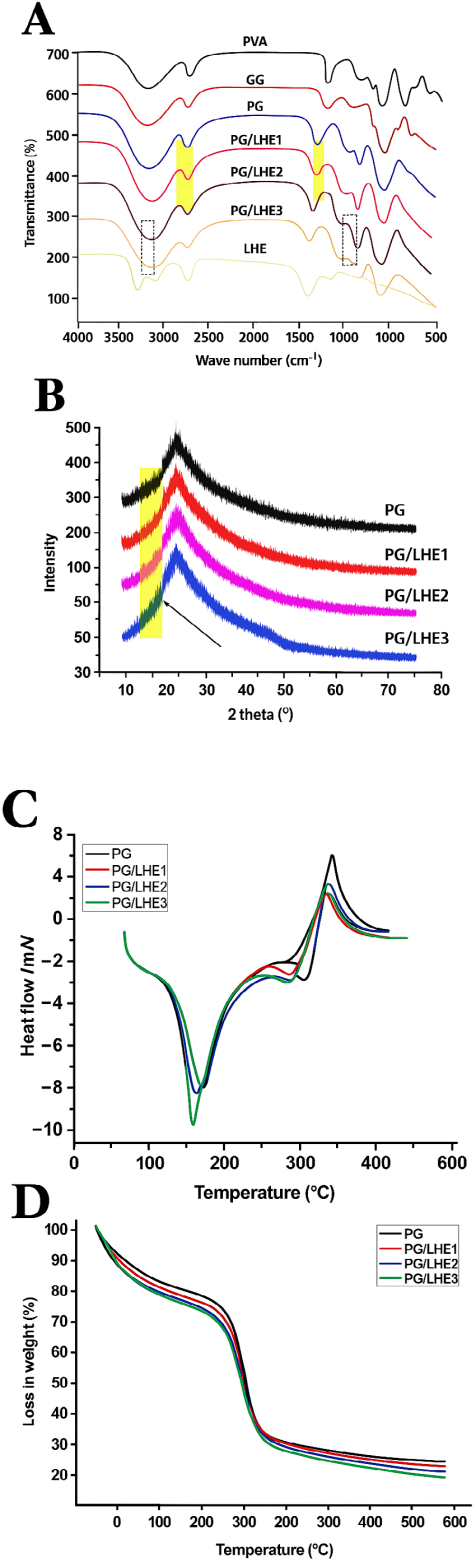
The films' FT‐IR transmittance (A), XRD patterns (B), DSC curves (C), and TGA thermograms (D) of PG/LHE films.

Following FT‐IR measurement, the relationship between the LHE and the PG film matrix was examined, as illustrated in Figure 2A. Concerning the PVA sample's FT‐IR spectra, Figure 2A reveals a broad, robust band centered at 3265 cm^−1^. This illustrates the robust hydrogen bonding hydroxyl group's stretching vibration. Furthermore, a narrower band at 2810 cm^−1^ is identified as stretching vibrations of CH_2_ and CH. Furthermore, the stretching vibrational band in the FT‐IR spectra at 1750 cm^−1^ is caused by the carbonyl functional groups. Based on the investigation, the C–O stretching vibration bands at 1050 and 1220 cm^−1^ were found to be bands susceptible to crystallization (Korbag and Mohamed Saleh 2016). The strong and broad absorption band at 3303 cm^− 1^ in the GG FT‐IR spectra was identified as the vibration caused by stretching the hydroxyl group on the sample's surface (Shi et al. 2008). The absorption peaks at 1013.7 and 2940 cm^−1^ correlate to bonds' bending and stretching vibrations of carbon dioxide and –CH_2_, respectively (Priyadarsini et al. 2022). Early absorption bands at 3253 cm^−1^ were produced by the chemical reaction between PVA and GG, and these bands were coupled with a significant absorption that suggests O–H bond stretching, which is linked to hydrogen bonding (Chatkitanan and Harnkarnsujarit 2020).

Furthermore, a delay in the absorption band's rise at 1742 cm^−1^ was noted. The band at 1022 cm^−1^ also demonstrates the existence of glycerol (–OH group) as a plasticizer (de Fernandes et al. 2022). The creation of H bonds between –COONa and ‐OH following the interaction between PVA and GG is most likely the cause of the reduction in strength and shifting of these distinctive absorption bands (Iqbal et al. 2020). The PG curves of the films remained unchanged when LHE was added at specified concentrations. This implies that the film's matrix can transport phenolic substances without undergoing any chemical modifications.

The FT‐IR spectrum of LHE exhibited distinct absorption bands that are consistent with the chemical structure of phenolic compounds. A broad band around 3400 cm^−1^ corresponded to O–H stretching vibrations, confirming the presence of hydroxyl groups commonly involved in hydrogen bonding. The absorption at ~2820 cm^−1^ was attributed to C–H stretching of aromatic and aliphatic moieties. A strong band near 1700 cm^−1^ indicated C=O stretching of carbonyl groups. Collectively, these characteristic bands verified the presence of hydroxyl, carbonyl, and aromatic functionalities, which are typical signatures of polyphenolic compounds such as flavonoids and phenolic acids. These findings align with the interpretation of polyphenolic structures reported previously (Naczk and Shahidi 2004).

Additionally, it was reported that PG and LHE interact physically, which is advantageous because it allows them to share the original active ingredients without compromising their bioactivity. Previous investigations have already confirmed the physical relationship (Gomaa et al. 2022). After adding 1.5% of LHE inside films, the spectral band of PG/LHE3‐2 shifted to 3218 cm^−1^, whereas PG/LHE3 showed a shift from 3253 cm^−1^. Furthermore, a spectral band shift was noted, going from 1050 cm^−1^ in PG/LHE1‐2 to 1110 cm^−1^ in PG/LHE3.

### 3.4 XRD Characteristics

XRD curves assessed the crystalline structure shape of PG films devoid of extracts and PG/LHE with various extract levels (Figure 2B). PG films and PG/LHE showed the usual patterns from the XRD study. It was discovered that adding plant extracts to the polymer films in the proper amounts did not affect the films' crystalline structure (Wang et al. 2019). Furthermore, at a Bragg angle of 2*θ* = 21.02°, the division in the primary peak in PG and PG/LHE1‐2 vanished in PG/LHE3. Therefore, including plant extracts with varying percentages disrupted the overall organization of the sustainable films to some extent (Chaudhary et al. 2021). Furthermore, it was noted that adding plant anthocyanin preparations might alter the films' diffraction peak height (Pourjavaher et al. 2017). Ensuring precise regulation of the quantity of herbal extract during the creation process is crucial for preserving the films' integrity, as the films' stability and usefulness depend heavily on their level of orderliness. The SEM photos, which demonstrated that films with greater concentrations of LHE (1.5%) had fissures in surface morphology, may be supported by the XRD data. Furthermore, the XRD results may also explain the drop in tensile strength and elongation at the break seen in the films. According to these results, the films' structure and physical characteristics are disrupted by a high concentration of LHE (1.5%), reducing their mechanical strength and flexibility.

### 3.5 Thermal Behavior of PG/LHE Films

Through DSC and TGA investigation, the thermal diagnostic of PG/LHE was shown in Figure 2C,D. DSC ascertained the temperature transition and variations in the crystallinity of the treated films (Riaz et al. 2020). Every sample showed unique endothermic and exothermic curves, as shown in Figure 2C. The water evaporation caused by the samples' heat absorption could cause the observed endothermic peak (Su et al. 2010).

As the temperature was raised to 350°C, PG/LHE films received enough energy to break down, resulting in an endothermic peak (Martins et al. 2012). Adding LHE to PG reduced the exothermic degree to 320.34°C, 312.22°C, and 308.43°C in PG/LHE1, PG/LHE2, and PG/LHE3, respectively. The control PG films showed an endothermic area in the range of 325.43°C. Because the incorporation of polyphenolic extracts led to modifications in the structure of the polymer matrix, the inclusion of LHE within PG films reduced their thermal stability. In addition, little energy is needed to degrade the polymer template, which may be explained by the structural alterations and disruption of crystallinity that occur when polyphenolic components are added to polymers (Gomaa et al. 2022; Qiao et al. 2017). Figure 2D illustrates a similar behavior in the thermal pattern of PG/LHE sheets utilizing thermal gravity analysis (TGA). In the first region, water is lost at temperatures between 65°C and 125°C, which causes the breakdown of –H bonds. The second stage, concerning carbonization and deterioration of leftover products following thermal degradation throughout phases, involves the depolymerization of PG polymers at temperatures between 225.62°C and 380.41°C. The results show that the mixing of LHE reduced the native film's thermal stability. The weight loss pattern shown in PG/LHE films indicates that the polymer chains' connection was weakened by the addition of LHE, which made the polymer easier to break down at lower temperatures. A similar tendency was noted earlier (Gomaa et al. 2022; Riaz et al. 2020).

### 3.6 Mechanical Properties of Produced Films

**FIGURE 3:**
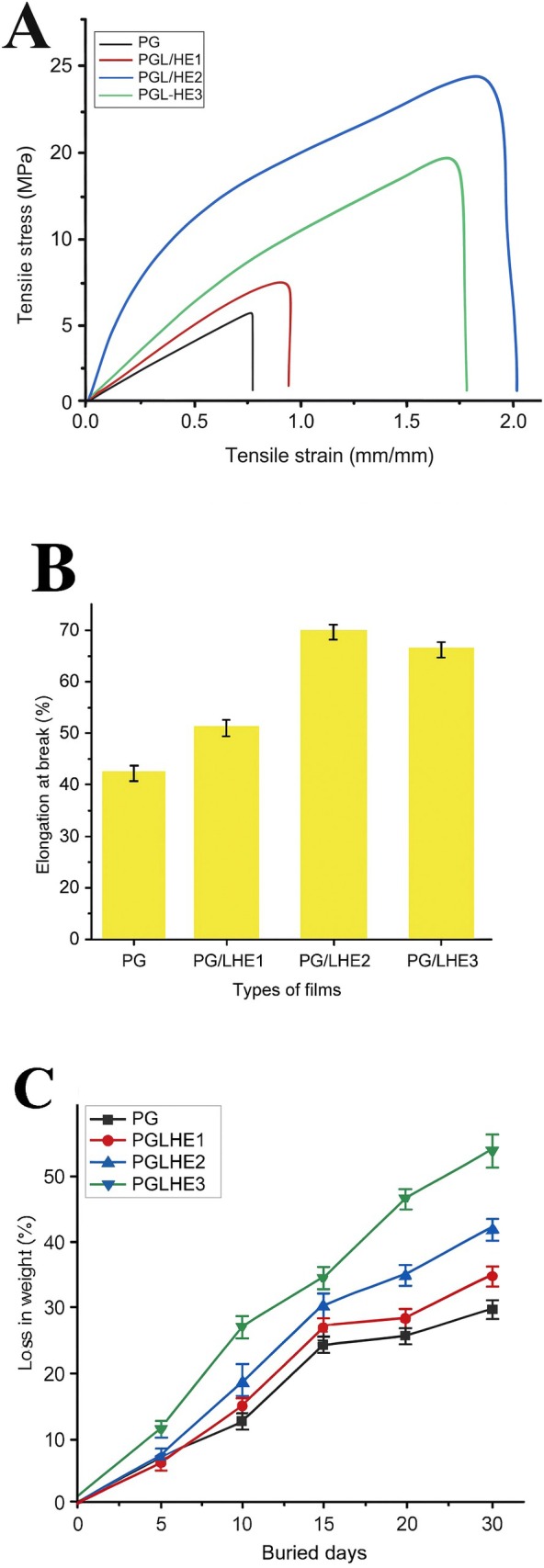
Tensile strength (A), elongation at break (B), and biodegradability (C) of PG/LHE films.

The mechanical characterizations of films can be shown by examining their elongation at break (EB), tensile stress (TS), and elastic modulus characteristics. As shown in Figure 3A and Table 2, the development of intermolecular hydrogen bonding between PVA, GG, and LHE in the presence of glycerol is responsible for the growing value of TS and aids in the plasticization and dispersion of both materials. The prior research clarified how the polyphenols from *Cinnamomum camphora* seeds and carboxymethyl chitosan‐gum Arabic enhanced TS and EB (Alnadari et al. 2022). When high concentrations of LHE 1.5% were used inside PG films, TS and EB stability decreased, leading to the loss of TS and EB features (Figure 3B). The mechanical properties of the film are greatly affected by a variety of crystalline properties in conjunction with the microstructure of the film network (Pastor et al. 2013). As a result, LHE was added to PG films at moderate concentrations of 1% to achieve the desired mechanical characterizations of the films. The neat PG film exhibited the lowest tensile strength (5.50 ± 0.13 MPa), elongation at break (31.13% ± 1.08%), and elastic modulus (22.09 ± 0.65 MPa), reflecting its relatively weak intermolecular interactions and poor elastic behavior. Upon incorporation of LHE polyphenols, all mechanical parameters improved markedly, with PG/LHE2 showing the highest tensile strength (22.23 ± 0.89 MPa), elongation at break (65.23% ± 0.97%), and modulus (62.86 ± 1.16 MPa), followed by PG/LHE3 and PG/LHE1 in descending order. The improvement can be attributed to strong hydrogen bonding and secondary interactions between the abundant hydroxyl groups of polyphenols and the hydroxyl‐rich PG matrix, which enhances chain compatibility, improves stress transfer, and reduces polymer chain slippage, thereby simultaneously increasing stiffness and extensibility. In contrast, the pristine PVA–guar gum (PG) film displayed low elasticity, likely because of poor compatibility and phase separation between the two polymer components, which restricts intermolecular interactions and cohesion, thereby limiting stress transfer and energy dissipation under tensile loading (Huang et al. 2023).

**TABLE 2:** Tensile strength, elongation at break, and elastic modulus of films.

Sample	Tensile strength (MPa)	Elongation at break (%)	Elastic modulus (MPa)
PG	5.50 ± 0.13^d^	31.13 ± 1.08^d^	22.09 ± 0.65^d^
PG/LHE1	7.20 ± 0.21 ^c^	42.62 ± 1.34 ^c^	28.80 ± 1.04 ^c^
PG/LHE2	22.23 ± 0.89 ^a^	65.23 ± 0.97 ^a^	62.86 ± 1.16 ^a^
PG/LHE3	16.41 ± 0.75 ^b^	61.34 ± 1.50 ^b^	45.71 ± 1.20 ^b^

*Note:* The values are expressed as the average ± standard deviation. The letters (a–d) in the same column indicate statistically substantial variations (*p* < 0.05) between the samples.

### 3.7 The Biodegradability of PG/LHE Films

Figure 3C shows the possible biodegradation outcomes of PG and PG/LHE films. As the days went by, there was a notable (*p* < 0.05) reduction in mass in PG/LHE films. After 30 days, the PG/LHE3 film had the highest degree of degradation, as seen by a reduction in weight of 45.87%; in contrast, PG films showed the lowest weight loss value, 26.16%. Plant extracts have been shown to accelerate the biodegradation of PG films in soil, fostering environmental sustainability. Investigations have indicated comparable results on incorporating other botanical extracts into films (Alnadari et al. 2022; Gomaa et al. 2022; Riaz et al. 2020).

### 3.8 Water Contact Angle (WCA) of PG/LHE Films

**FIGURE 4:**
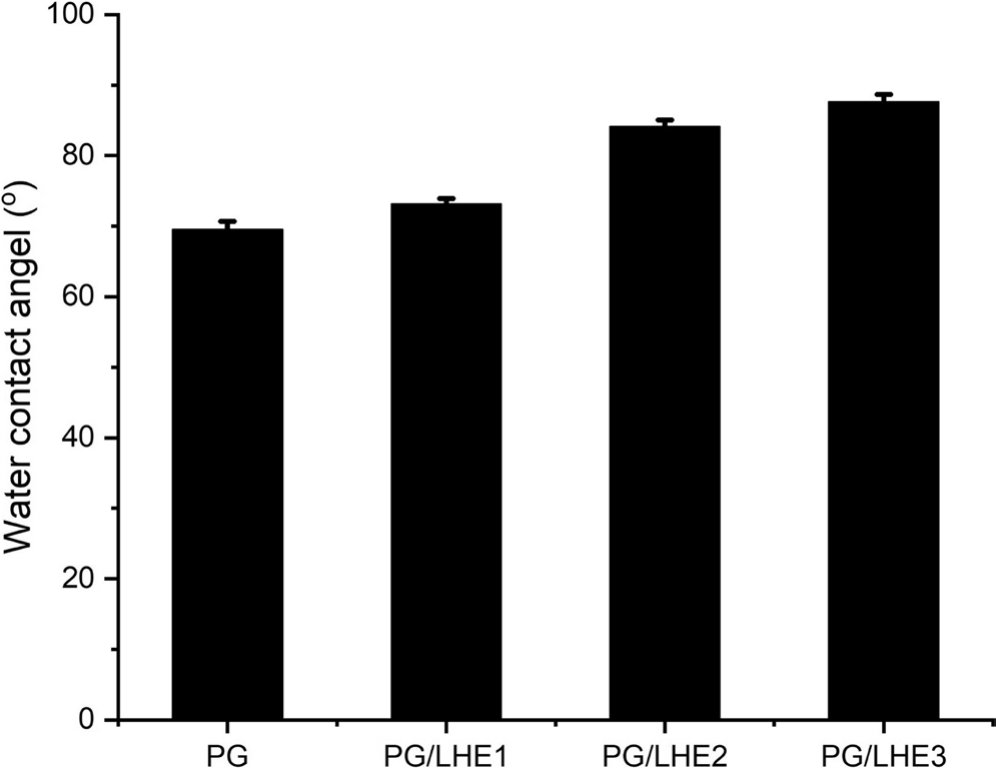
Water contact angle of PG/LHE films.

WCA is frequently measured to determine whether a surface is hydrophilic or hydrophobic. A surface's degree of surface activation can be assessed by observing the angle at which water droplets converge on it. As indicated in Figure 4, the PG film exhibited a WCA of 69.5°, indicating a hydrophilic surface due to the presence of abundant hydroxyl groups. Incorporation of *Lotus halophiles* extract (LHE) progressively increased the WCA, reaching 87.8° in PG/LHE3. This enhancement is attributed to the interaction of hydrophobic polyphenolic compounds with the PG matrix, which reduces surface polarity and increases water resistance, thereby improving the films' suitability for food packaging applications (Abdin et al. 2023). Additionally, polyphenols such as tannins or gallic acid derivatives contain phenolic rings that can diminish the polar character of the film surface, favoring dispersion interactions over polar ones. This lowers surface energy and reduces wettability, thus increasing the WCA. Similar effects were observed when hydrophobic plant‐derived compounds were incorporated into biopolymeric films, leading to enhanced water resistance (Mathew et al. 2025).

### 3.9 Antioxidant Activity of PG/LHE Films

**FIGURE 5:**
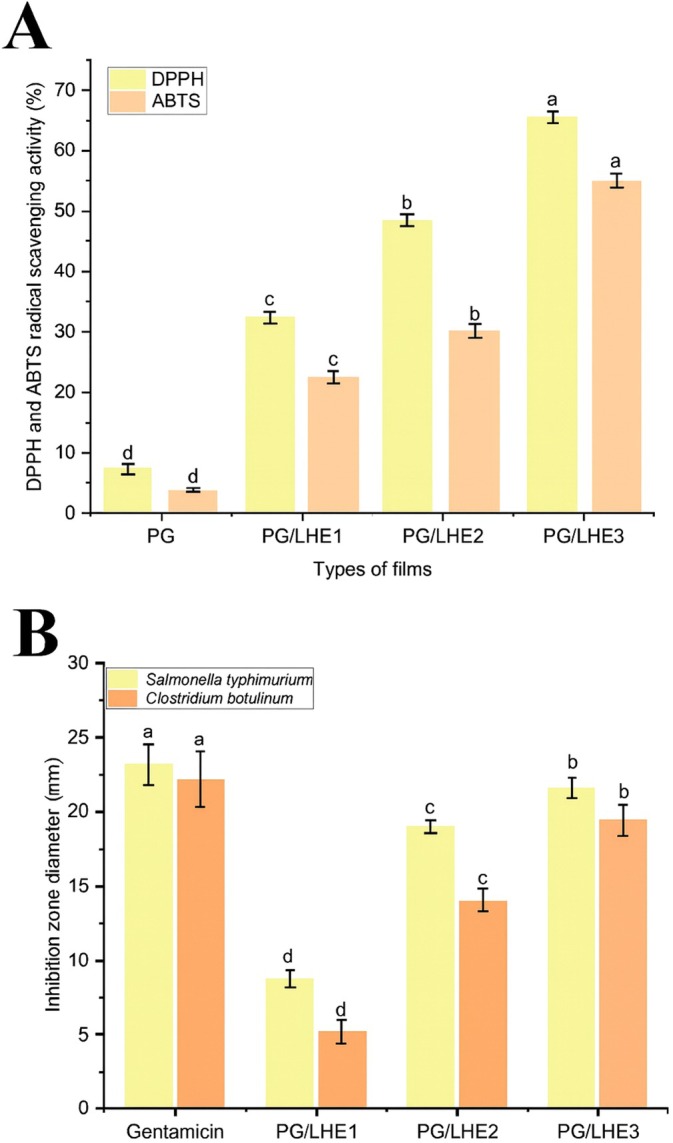
The antioxidant activity (A) and antibacterial effect (B) of PG/LHE films. The distinct letters inside the bars of the identical hue for figures (A and B) denote noteworthy disparities.

As shown in Figure 5A, the findings proved that PG/LHE3 films had the maximum value of scavenging activity (67.16% and 55.19%) for DPPH and ABTS radicals, respectively, followed by PG/LHE2 and PG/LHE4 (*p* < 0.05). According to the study, extracts are primarily responsible for the films' antioxidant capacity. This is probably due to the extracts' high hydroxyl group content, which may serve as a source of hydrogen for DPPH and ABTS radicals (Mittal et al. 2021). The enhanced antioxidant capacities of the PG/LHE films, particularly PG/LHE3, can be directly attributed to the rich profile of phenolic compounds in the LHE. Phenolic acids such as ferulic acid exhibit strong radical‐scavenging activity, especially against ABTS radicals as they donate hydrogen atoms to stabilize free radicals and lower oxidative stress (Petretto et al. 2017). Meanwhile, flavonoids like quercetin, kaempferol, and their glycosides are highly effective antioxidants in both DPPH and ABTS assays due to their conjugated aromatic structures and hydroxyl groups capable of delocalizing unpaired electrons. Packaging films with improved antioxidant properties is one method for increasing food products' shelf life that shows promise and works well. The antioxidant activity from adding extracts with high hydroxyl group content to the films might help stop or slow food product oxidation, minimizing spoiling and increasing the packaged goods' shelf life.

### 3.10 Antibacterial Activity of PG/LHE Films

Antibacterial agents control bacteria to prevent food spoilage and foodborne illnesses by inhibiting toxin production. The investigated samples exhibited inhibition zone sizes ranging from 9.17 to 20.15 mm against *S. typhimurium* , and from 5.15 to 18.12 mm against *C. botulinum* , as indicated in Figure 5B. The tested films exhibited broad‐spectrum antibacterial activity that was most successful when it came to the PG/LHE3 films. The films' respective inhibition zones measured 20.15mm for *S. typhimurium* and 18.12 mm for *C. botulinum*. Consequently, the tested films' LHE content had the most pronounced inhibitory effect on bacterial development. Active ingredients in extracts can potentially modify the permeability of bacterial cell walls and cell membranes. This phenomenon may be elucidated by the hydrogen bonds that develop between enzymes and bioactive substances (Cierpiał et al. 2020). The antibacterial properties of plant extracts followed by the family *Fabaceae* were provided previously (Obistioiu et al. 2021).

The antibacterial effects of PG/LHE films against *S. typhimurium* and *C. botulinum* can also be explained by the diverse bioactive compounds present in LHE. Polyphenols such as quercetin, kaempferol, and tamarixetin exert antimicrobial action through disruption of bacterial cell membranes, inhibition of nucleic acid synthesis, and interference with energy metabolism, making them effective against Gram‐negative pathogens such as Salmonella (Cushnie and Lamb 2011). In addition, phenolic acids, including ferulic acid and gallic acid, contribute to antibacterial efficacy by lowering intracellular pH, altering membrane permeability, and chelating essential ions, which disrupt enzyme systems in both Gram‐positive and Gram‐negative bacteria (Borges et al. 2013). Flavonoids like genistein and astragalin further enhance this effect through synergistic interactions that impair microbial adhesion and biofilm formation, mechanisms especially relevant against spore‐forming bacteria like *C. botulinum* . The relatively high concentrations of ferulic acid (71.42 μg/g), genistein (61.71 μg/g), and gallic acid (43.36 μg/g) in LHE thus provide a strong molecular basis for the observed inhibitory activity of PG/LHE films. Collectively, these findings suggest that the incorporation of LHE into PG films not only strengthens antioxidant defenses but also imparts broad‐spectrum antibacterial properties, enhancing their potential in active food packaging.

### 3.11 Physical Characteristics of PG/LHE Films

The physical properties of PG films may change if active ingredients like LHE are added. Table 3 shows that the thickness of the edible film rose considerably (*p* < 0.05) when the LHE content in PG films increased. For PG/LHE3, the greatest thickness of 0.164 mm was noted. A reasonable hypothesis is that there is a positive relationship between the polyphenols' content and the films' thickness. The polyphenols often cluster together to create a mesh‐like arrangement within the film, giving the impression that the film is thicker (Sogut and Seydim 2018).

**TABLE 3:** Physical characteristics of PG sheets enhanced using LHE.

Films samples	Thickness	Swelling degree (%)	Solubility (%)	Moisture content (%)	*WVP* (× 10^−10^ g.m^−1^ s^−1^ pa^−1^)
PG	0.106 ± 0.0030^d^	36.22 ± 0.17^a^	25.41 ± 0.84^a^	22.18 ± 0.27^a^	4.123 ± 0.004^b^
PG/LHE1	0.127 ± 0.0022^c^	32.43 ± 0.21^b^	21.22 ± 0.03^b^	15.14 ± 0.35^b^	3.115 ± 0.002^c^
PG/LHE2	0.143 ± 0.0023^b^	25.09 ± 0.32^c^	18.71 ± 0.40^c^	12.31 ± 0.19^c^	2.121 ± 0.004^d^
PG/LHE3	0.164 ± 0.0013^a^	21.71 ± 0.40^d^	15.62 ± 0.37^d^	9.87 ± 0.14^d^	4.731 ± 0.001^a^

*Note:* The values are expressed as the average plus or minus the standard deviation. The letters (a–d) in the same column indicate statistically substantial variations (*p* < 0.05) between the samples.

The findings in Table 3 demonstrate that the films' degree of swelling and solubility reduced up to 21.71% and 15.62%, respectively, as the concentration of polyphenols in the films rose. Many polyphenols are hydrophobic, indicating their limited solubility in water. This characteristic causes them to cluster together, forming a network that reduces the solubility and swelling of the film. Additionally, the degree of swelling and water solubility can provide insights into the hydrophilicity of the film's components. A film's optimal water solubility level can differ based on its intended application. When the film is utilized to package extremely wet meals, it may need to be less soluble to maintain its structure. However, if the film is intended to be consumed alongside the food, it may be better to be more soluble (Alves et al. 2018).

Regarding moisture content, a rise in LHE content reduces the film's moisture content. This occurs because the functional groups in LHE are hydrophobic and do not attract water. The network created by these hydrophobic groups inhibits water molecules from penetrating the film. Furthermore, including LHE may restrict water contact by enhancing the intermolecular bonding between hydroxyl/amino groups and phenolics within the polymer matrix.

A similar characteristic was previously noted following the integration of plant extracts into gum Arabic films (Gomaa et al. 2022). The *WVP* values for PG and PG/LHE are shown in Table 3. According to the study, PG films' water vapor transfer rate was 2.234 to 1.231 × 10^−10^ g.m^−1^ s^−1^ pa^−1^. The *WVP* for PG/LHE1‐2 films decreased somewhat from 3.115 to 2.121 × 10^−10^ g.m ^−1^ s^−1^ pa^−1^ (*p* < 0.05) when the LHE loading rose from 0.5% to 1%. These results align with earlier research that proposes that the extracts' phenolic groups may form bonds with polymer molecules to stop those groups from mixing with water and eventually lower the *WVP* (Chaudhary et al. 2021; Severo et al. 2021). Nonetheless, the steady addition of 1.5% concentration of LHE led to a rise in *WVP* to 4.731 × 10^−10^ g.m^−1^ s^−1^ pa^−1^. Water vapor evaporating from films may be explained by the internal fissures and agglomerates on the surface that developed after utilizing larger concentrations from LHE.

### 3.12 Color Characteristics and Opacity of PG/LHE Films

Table 4 shows that the opacity and color index of the film samples made with various mixes of PG and LHE additives varied significantly. As the amount of LHE rises, the samples' L* value falls. PG films exhibited the highest L* value (93.53 ± 0.542), whereas PG/LHE3 displayed the lowest L* value (28.67 ± 0.154). Up to 1% (PG/LHE2), the samples' a* and b* values rise as the concentration of LHE additives increases. PG/LHE2 films had the highest a* value (8.72), while PG films had the lowest a* value (0.09). PG/LHE2 films had the highest b* value (43.58), while PG films had the lowest b* value (2.19). The principal extract's color caused the color shifts while using various LHE concentrations. PG/LHE3 had the highest opacity value (4.132), while PG showed the lowest (1.452). These findings imply that opaque films with maximum a* and b* values can be produced using PG/LHE2 films, which may be useful for protecting light‐sensitive goods such as fatty food items.

**TABLE 4:** Color characteristics of PG films combined with LHE.

Films samples	L*	a*	b*	∆*E*	Opacity
PG	93.53 ± 0.542^a^	0.09 ± 0.144^d^	2.19 ± 0.234 ^d^	18.234 ± 0.221^d^	1.452 ± 0.022^d^
PG/LHE1	75.40 ± 0.264^b^	4.84 ± 0.351^c^	37.15 ± 0.273^b^	22.54 ± 0.176^c^	2.9876 ± 0.023^c^
PG/LHE2	52.29 ± 0.243^c^	8.72 ± 0.262^a^	43.58 ± 0.312^a^	61.65 ± 0.761^b^	3.424 ± 0.052^b^
PG/LHE3	28.67 ± 0.154^d^	7.38 ± 0.211^b^	13.24 ± 0.252^c^	93.76 ± 0.487^a^	4.132 ± 0.016^a^

*Note:* The values are expressed as the average plus or minus the standard deviation. The letters (a–d) in the same column indicate statistically substantial variations (*p* < 0.05) between the samples.

### 3.13 Application of PG/LHE2 on Olive Oil

**FIGURE 6:**
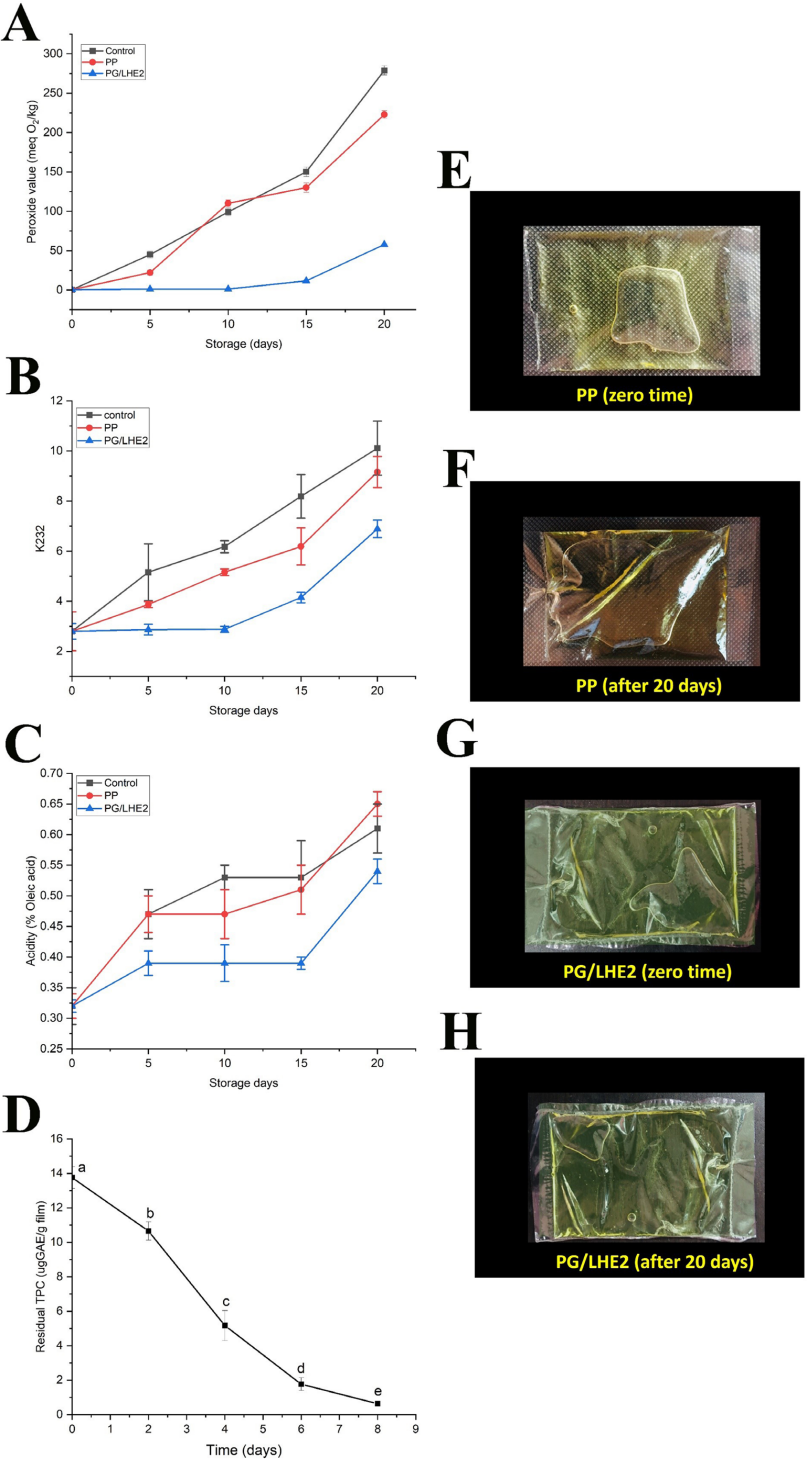
Peroxide value (A), K_232_ value (B), acidity (C), residual TPC content of polyphenolic compounds in PG/LHE2 films (D), and types of packaging films (E–H) on olive oil during storage.

The research evaluated the impacts of utilizing *PG/LHE2* film as a wrapper material for olive oil and compared it with using uncoated oil (control) and polypropylene (PP) cover (Figure 6D–G). Figure 6A shows olive oil's peroxide value (PV) covered in various materials. The control and PP packaged samples significantly rose in peroxide values after 5 days of storage. However, the PG/LHE2 packaged samples demonstrated remarkable stability until the fifteenth day. Therefore, the oxidative stability of olive oil throughout storage may benefit from the active PG/LHE2 films. Antioxidants present in bio‐composite sheets prevent greasy products from oxidizing too quickly and maintain their quality over time (Flórez et al. 2022; Lyn et al. 2021; Stoll et al. 2017). As a quality indicator, the absorption coefficient K232 measures absorbance at 232 nm, linked to conjugated dienes and hydroperoxides, indicating an intermediate and early oxidation stage, respectively (Al‐Smadi et al. 2014). The PG/LHE2 film demonstrated the best performance in shielding olive oil from the oxidative reaction during the K232 findings out of all the treatments (Figure 6B). After 5 days of storage under rapid deterioration, the olive oil packaged in PP and the control sample surpassed the permitted limit stipulated by the Codex Alimentarius (K232 ≤ 2.5).

In contrast, the oil packaged in PG/LHE2 films remained within the limit and began to increase after 10 days of accelerated conditions. Carboxyl and hydroxyl bonds in the film substrate suggest that PG/LHE2 films have sufficient antioxidant activity to safeguard the butter samples from subsequent oxidation, allowing for an extended food storage period. The same results were obtained earlier (Candido et al. 2022). Assessing acidity is essential for ascertaining the quality of oil‐based products. The water‐induced hydrolysis of triglycerides increases free fatty acids within oils and fats, elevating their acidity and potentially degrading the overall quality. This, in turn, can accelerate both oxidation and the onset of rancidity. The data shown in Figure 6C indicates that, after 15 days of storage, the acidity content of samples packed with PG/LHE2 did not significantly alter. However, during the 5 days, the acidity level of the PP and the control significantly increased. There are multiple reasons for this. First off, the films' reduced moisture content due to the presence of LHE restricts the amount of water that can reach the triglycerides, which in turn lessens the hydrolysis of the triglycerides. An additional plausible explanation could be the reduced *WVP* of the active films, as demonstrated in Section 3.11, and the increased WCA, as mentioned in Section 3.7, which lessens the transfer of moisture through the films. The outcomes followed the same general pattern as the earlier results (Marand et al. 2021). Films in oil packaging demonstrate how effectively the materials block oxygen. When PVA films were combined with other polymers and components, the size and number of PVA crystallites diminished. However, this increased crosslinking density causes tighter molecular packing and reduced free volume in the noncrystalline area. As a result, this could help delay oxygen penetration (Lim et al. 2015). Ensuring the quality of olive oil is preserved indirectly signifies that the packaging has a low permeability to oxygen (Carpiné et al. 2015).

### 3.14 Release of Polyphenolic Compounds From PG/LHE2 Sachets to Olive Oil

Figure 6D shows a controlled release of phenolic compounds from the PG/LHE2 film into the olive oil, with the residual antioxidant content in the film decreasing from a high of approximately 13.6 μg GAE/g at Day 0 to a low of nearly 0.89 μg GAE/g by Day 8. This sustained release over the 8 days is crucial for preserving olive oil quality as it continuously replenishes the oil's antioxidant defense system, actively scavenging free radicals to delay the onset of rancidity and oxidation, thereby significantly extending the shelf life and maintaining the oil's sensory and nutritional properties. This controlled release is paramount for quality preservation, as the migrated phenolic compounds directly combat lipid oxidation, the primary cause of olive oil rancidity. These antioxidants scavenge free radicals and interrupt autoxidation chain reactions (Frankel 2008). The obtained results confirm the ability of films to maintain the quality of olive oil during storage days, as explained in Figure 6A–C.

## 4 Conclusions

This research successfully developed new bio‐composite films using PG and LHE. The findings revealed that including LHE at a concentration of up to 1% in PV/CM films resulted in a reduction in *WVP* up to 2.121 × 10^−10^ g.m^−1^ s^−1^ pa^−1^, solubility up to 18.71%, swelling degree up to 25.09% and moisture content up to 12.31% indicating that the films were effective in maintaining optimal moisture levels around packaged products. Moreover, the films exhibited improved water contact angle, antioxidant activity, opacity, and antibacterial activity. LHE inside PG films (PG/LHE2) enhanced the tensile strength and elongation at break properties. When tested on olive oil, the PG/LHE2 packaging film demonstrated a delay in decreasing quality characteristics during accelerated storage conditions by stabilizing PV content, K_232_, and acidity up to 15 days of storage, outperforming traditional plastic packaging films. In conclusion, these biodegradable films have the potential to serve various food applications, offering benefits for prolonged shelf life.

## Author Contributions

**Osama Magouz:** conceptualization, study design, supervision, and drafting of the manuscript; **Ahmed Aboueloyoun Taha** and **Diaeldin Omer Abdelkarim:** conceptualization, study design, and data acquisition; **Mohamed Abdin:** conceptualization, study design, data acquisition, data analysis and interpretation, drafting of the manuscript, and sample collection. **Eman Fayed, Khadija S. Radhi**, and **Fayez Althobaiti:** methodology, investigation, design, and execution of analysis, contribution of data and analytical tools, and review of the final manuscript; **Reham M. Kamel:** conceptualization, study design, supervision, drafting of the manuscript, and sample collection; **Yahya S. Hamed:** data interpretation, manuscript drafting, and sample collection; **Mahmoud Younis** and **Lamiaa I. El‐Nawasany:** conceptualization, study design, supervision, and manuscript drafting.

## Conflicts of Interest

The authors declare no conflicts of interest.

## Data Availability

Data will be made available on reasonable request.
