# Correction to “Eco‐Friendly Innovation: Biodegradable and Oil‐Resistant Bags From *Lotus halophilus* Extract, Polyvinyl Alcohol, and Guar Gum for Sustainable Food Packaging”

**DOI:** 10.1002/fsn3.71560

**Published:** 2026-02-15

Magouz, O., E. Fayed, K. S. Radhi, et al. 2025. “Eco‐Friendly Innovation: Biodegradable and Oil‐Resistant Bags From *Lotus halophilus* Extract, Polyvinyl Alcohol, and Guar Gum for Sustainable Food Packaging.” *Food Science and Nutrition* 13, no. 10: e71017. https://doi.org/10.1002/fsn3.71017.

Description of corrections:

In the originally published version, the following errors are noted:

The co‐author's name “Eman Fayed” was incorrectly spelled. The correct spelling is “Eman Fayad.”

The affiliation of Fayez Althobaiti was incorrectly listed as

^3^Department of Science and Nutrition, College of Science, Taif University, Taif, Saudi Arabia.

It should be corrected to:

^2^Department of Biotechnology, College of Sciences, Taif University, Taif, Saudi Arabia.”

The online version has been corrected accordingly.

We apologize for these errors.

